# The crucial role of particle surface reactivity in respirable quartz-induced reactive oxygen/nitrogen species formation and APE/Ref-1 induction in rat lung

**DOI:** 10.1186/1465-9921-6-129

**Published:** 2005-11-02

**Authors:** Catrin Albrecht, Ad M Knaapen, Andrea Becker, Doris Höhr, Petra Haberzettl, Frederik J van Schooten, Paul JA Borm, Roel PF Schins

**Affiliations:** 1 Institut für Umweltmedizinische Forschung (IUF) at the Heinrich-Heine-University Düsseldorf, Germany; 2 Nutrition and Toxicology Research Institute Maastricht (NUTRIM), Department of Health Risk Analysis and Toxicology, University of Maastricht, The Netherlands

## Abstract

Persistent inflammation and associated excessive oxidative stress have been crucially implicated in quartz-induced pulmonary diseases, including fibrosis and cancer. We have investigated the significance of the particle surface reactivity of respirable quartz dust in relation to the *in vivo *generation of reactive oxygen and nitrogen species (ROS/RNS) and the associated induction of oxidative stress responses in the lung. Therefore, rats were intratracheally instilled with 2 mg quartz (DQ12) or quartz whose surface was modified by either polyvinylpyridine-N-oxide (PVNO) or aluminium lactate (AL). Seven days after instillation, the bronchoalveolar lavage fluid (BALF) was analysed for markers of inflammation (total/differential cell counts), levels of pulmonary oxidants (H_2_O_2_, nitrite), antioxidant status (trolox equivalent antioxidant capacity), as well as for markers of lung tissue damage, e.g. total protein, lactate dehydrogenase and alkaline phosphatase. Lung homogenates as well as sections were investigated regarding the induction of the oxidative DNA-lesion/oxidative stress marker 8-hydroxy-2'-deoxyguanosine (8-OHdG) using HPLC/ECD analysis and immunohistochemistry, respectively. Homogenates and sections were also investigated for the expression of the bifunctional apurinic/apyrimidinic endonuclease/redox factor-1 (APE/Ref-1) by Western blotting and immunohistochemistry. Significantly increased levels of H_2_O_2 _and nitrite were observed in rats treated with non-coated quartz, when compared to rats that were treated with either saline or the surface-modified quartz preparations. In the BALF, there was a strong correlation between the number of macrophages and ROS, as well as total cells and RNS. Although enhanced oxidant generation in non-coated DQ12-treated rats was paralleled with an increased total antioxidant capacity in the BALF, these animals also showed significantly enhanced lung tissue damage. Remarkably however, elevated ROS levels were not associated with an increase in 8-OHdG, whereas the lung tissue expression of APE/Ref-1 protein was clearly up-regulated. The present data provide further *in vivo *evidence for the crucial role of particle surface properties in quartz dust-induced ROS/RNS generation by recruited inflammatory phagocytes. Our results also demonstrate that quartz dust can fail to show steady-state enhanced oxidative DNA damage in the respiratory tract, in conditions were it elicits a marked and persistent inflammation with associated generation of ROS/RNS, and indicate that this may relate to compensatory induction of APE/Ref-1 mediated base excision repair.

## Background

Worldwide, millions of people are occupationally exposed to crystalline silica (e.g. quartz) dust. Chronic exposure to quartz can lead to a variety of pulmonary diseases, including silicosis and cancer [1]. Notably however, studies on quartz-induced carcinogenicity have revealed that the quartz hazard is a variable entity [2], as carcinogenic outcomes seem to be inconsistent and show a rather large variation [1]. Indeed, the toxicity of quartz is highly variable and has been demonstrated to largely depend on the reactivity of its particle surface [3]. Currently, there is a large body of experimental evidence showing that modification of the particle surface by either grinding or coating with PVNO or aluminium salts modifies quartz-induced cytotoxicity, genotoxicity, inflammogenicity and fibrogenicity [4-11].

It is now generally accepted that excessive and persistent formation of ROS and RNS plays a major role in quartz-induced silicosis and carcinogenicity [3,12-14]. During quartz exposure, ROS may be generated via two major routes: either from the quartz particles themselves, or indirectly, from the oxidative burst of pulmonary inflammatory cells (i.e. neutrophils and macrophages) that invade the lung upon exposure to quartz. Previously, we and others have demonstrated that the surface characteristics of quartz are involved in both of these pathways, since surface-modification significantly impacts on the generation of ROS by quartz particles in an acellular environment [6,15], as well as on the induction and persistence of pulmonary inflammation [4,7,9,11]. It has been also demonstrated that such quartz-surface modifications directly modify the release of ROS from neutrophils and macrophages upon *in vitro *treatment with quartz [6,16-18]. Notably however, current evidence for a role of the reactive particle surface on the actual generation of ROS *in vivo *and the oxidative stress response in lung tissue are merely associative.

In the context of the inflammation-mediated carcinogenic effects of quartz, it should be noted that ROS are on the one hand known to activate redox-sensitive signal transduction cascades, such as nuclear factor kappa B (NFκB) and activator protein (AP-1), which are involved in activation of genes controlling inflammation, proliferation and/or apoptosis [11]. On the other hand, quartz-mediated formation of ROS is considered to drive oxidative DNA damage and associated mutagenesis [13,19]. The importance of inflammation-mediated ROS for quartz mutagenesis was initially provided by Driscoll and co-workers [20]. Using a complementary *in vivo *and *ex vivo/in vitro *approach, they showed (a) that quartz particles are mutagenic to rat lung epithelial cells *in vivo *in association with a persistent pulmonary inflammation, and (b) that BAL cells obtained from such quartz-treated rats are mutagenic to rat lung epithelial cells *in vitro*. In concordance with these observations, quartz has been shown to induce the premutagenic oxidative DNA adduct 8-OHdG in rat lungs [21,22]. In an *in vitro *co-incubation model we could then demonstrate that the induction of 8-OHdG in lung epithelial cells by neutrophils can be blocked by antioxidants [23].

To cope with exogenous DNA damage, e.g. as may be induced upon quartz exposure, cells are equipped with multiple DNA repair enzymes. The repair of oxidative DNA lesions such as 8-OHdG, predominantly occurs via the base excision repair (BER) pathway. As such, altered expression of BER enzymes has been proposed as a sensitive marker of the induction of oxidative stress and oxidative DNA damage [24,25]. Among these repair proteins, the expression of the bifunctional protein APE/Ref-1 represents a highly interesting candidate [25]. The protein APE/Ref-1 consists of two functionally independent domains, the highly conserved C-terminus, involved in both the short-patch and long-path pathways of BER, and the completely unconserved N-terminus, which exerts the control of several redox-sensitive transcription factors including NFκB and AP-1. Previously, *in vitro *studies have shown that asbestos fibres enhance APE/Ref-1 expression in mesothelial cells as well as in alveolar macrophages, which has been linked to its role in oxidative DNA damage repair and ROS-mediated regulation of redox-sensitive transcription pathways, respectively [26,27]. So far, however, the role of quartz-elicited ROS generation on APE/Ref-1 expression *in vivo *has not been elucidated.

The aim of our present study was to investigate whether inhibition of the surface reactivity of quartz particles modulates inflammation-mediated generation of ROS and RNS in the rat lung *in vivo*, and whether this impacts on pulmonary toxicity and more specifically, on the expression of the lung tissue sensors of oxidative stress/DNA damage 8-OHdG and APE/Ref-1. Therefore, BAL as well as lung tissue from rat lungs were analysed for pulmonary toxicity, inflammation, ROS/RNS generation and induction of 8-OHdG and APE/Ref-1, seven days after a single instillation with native quartz or quartz from which the surface was coated with either PVNO or AL.

## Methods

### Chemicals

2-2'-azinobis-(-3 ethylbenzothiazoline-6-sulphonate) (ABTS), dimethyl sulphoxide (DMSO), ethidium bromide, L-glutamine, Ham's F12 medium, Hank's balanced salt solution (HBSS), HEPES buffer, fetal calf serum (FCS), penicillin/streptavidin solution, phosphate buffered saline (PBS), were all obtained from Sigma (Germany). Horseradish peroxidase (HRP), guaiacol, phorbol-12-myristate-13-acetate (PMA), anti-mouse-IgG whole protein HRP-conjugated secondary antibody and the tubulin antibody as well as Diaminobenzidine were also purchased from Sigma (Germany). ABAP (2,2'-azobis-(-2-amidinopropane)HCl was from Polysciences, Warrington, USA. F12-K Nutrient Mixture was obtained from Invitrogen (Germany). Protease inhibitors in form of a Complete™ cocktail were purchased from Roche Diagnostics GmbH (Germany). The Bradford-protein assay was used from BioRad (Germany). ECL-reagent/detection system was obtained from Amersham Bioscience (Germany). The antibody against 8-OHdG was obtained from the Institute of Aging (Japan) and the antibody against APE/Ref-1 (C-4) was purchased from Santa Cruz Biotechnology (USA). For immunohistochemical detection secondary biotinylated horse-anti mouse antibody, the streptavidin-biotin-system (Vectastain *Elite *Kit) and mouse IgG were used from Vector Laboratories (USA). DePex was used from Serva (Germany). Hoechst 33342 was obtained from Sigma (Germany), and MFP488 goat anti-mouse IgG from MoBiTec (Germany). All other chemicals were from Merck (Germany) and were of highest purity.

### Surface treatment of quartz

Surface modification of Quartz (DQ12, batch 6, IUF, Düsseldorf) was performed as described previously [10]. Briefly, DQ12 was coated for 5.5 hours in a 5 mg/ml suspension in a 1% dilution of either PVNO or AL, dissolved in distilled deionised sterile water. Non-coated DQ12 was suspended in water without any further additions. After repetitive washings in sterile water, the particles were finally resuspended in sterile water at a concentration of 5 mg/ml in sterile glass tubes, and allowed to dry under a laminar flow chamber. All quartz processing was performed under sterile conditions, and a single batch of non-coated and coated quartz was prepared and used for the whole study to avoid possible variable coating efficiency. Atomic absorption spectrometry and spectrophotometry revealed coating efficiencies of respectively 11 μg PVNO/mg quartz and 1.6 μg aluminium/mg quartz, and transmission electron microscopy analysis showed that the coating procedures did not cause changes in particle size distribution or aggregation of the DQ12 [11]. For intratracheal (i.t.) instillation, the dried quartz preparations were resuspended in 1 ml of PBS (without Mg^++ ^and Ca^++^) and sonicated in a water bath (Sonorex TK52; 60 Watt, 35 kHz, 5 min).

### Quartz instillation and bronchoalveolar lavage

Specific pathogen free female Wistar rats were used for the study (Janvier, Le Genest St. Isle, France). The animals were housed and maintained in an accredited on-site testing facility, according to the guidelines of the Society for Laboratory Animals Science (GV-SOLAS). Food and water were available *ad libitum*. When weighing 200–250 g (8 weeks old), animals were anaesthetised with isoflurane and i.t. instillation was performed using a laryngoscope. From the non-coated or coated quartz suspensions (5 mg/ml in PBS) 400 μl were instilled giving a final dose of 2 mg per rat (n = 5 per treatment and endpoint). Control rats were instilled with only PBS. Separate control animals received 22 μg PVNO or 35 μg AL (in PBS), amounts calculated from the coating efficiency of both substances, to assess possible direct effects of coating materials. After 7 days, animals were sacrificed by a single i.p. injection of Na-pentobarbital and subsequent exsanguination via the abdominal aorta. The lung was cannulated via the trachea and BAL was performed *in situ *by infusing the lungs with 5 ml aliquots of PBS. The BALF was drained passively by gravity and the procedure was repeated four times, giving a total BAL volume of 20 ml. Total cell number in the BAL was analysed using a hemocytometer chamber (Neubauer) and viability was assessed by trypan blue dye exclusion. BAL-cell differential was determined on cytospin preparations stained with May-Grünwald/Giemsa (MGG). The BALF was centrifuged twice (300 g to collect cells, followed by 1000 g to obtain BALF), and the cell-free supernatant was analysed for lung injury parameters, e.g. total protein, LDH and AP, as well as myeloperoxidase (MPO).

### Measurement of cytotoxic and inflammogenic bronchoalveolar lavage parameters

Total protein was analysed according to the method described by Lowry. LDH and AP were assayed using diagnostic kits from Merck (Germany). MPO activity in the BALF was assayed according to the method originally described by Klebanoff et al [28]. Briefly, 200 μl of cell-free BALF was mixed with 800 μl MPO assay solution, containing 107.6 ml H_2_O, 12 ml 0.1 M sodium phosphate buffer, 0.192 ml Guaiacol, 0.4 ml 0.1 M H_2_O_2_. The generation of tetra-guaiacol was measured spectrophotometrically (Beckman) at 470 nm and the change of optical density per minute was calculated from the initial rate. The MPO activity was calculated from the formula: U/ml = ΔOD/minute × 0.752 and expressed as mU/ml. One unit of the enzyme is defined as the amount that consumes 1 μmol H_2_O_2 _per minute.

### Measurement of hydrogen peroxide and nitrite in bronchoalveolar lavage fluid

Freshly obtained BALF was used to measure hydrogen peroxide according to the method of Gallati and Pracht [29]. Therefore, 75 μl of BALF was mixed with 75 μl of a 3,3',5,5'-Tetramethylbenzidine solution (TMB solution EIA, solution A, Bio-Rad), containing 8.5 U/ml horseradish peroxidase. After 10 min incubation at RT, 50 μl H_2_SO_4 _(1 M) was added and absorption was measured at 450 nm using a microplate reader (Multiskan, Labsystems). The final concentration of H_2_O_2 _was calculated from a standard curve made up in BALF obtained from an untreated rat.

Nitric oxide levels in BALF were determined by analysis of its relative stable metabolite nitrite using the Griess reaction. Briefly, 100 μl of the cell free BALF was incubated with an equal volume of Griess reagent (0.1% naphthylene ethylene-diamide.2HCl, 1% sulfanilamide, 2.5% phosphoric acid) at room temperature for 10 minutes. Absorbance (540 nm) was then determined using a microplate reader and concentrations were calculated from a NaNO_2 _standard curve.

### Measurement of ex vivo hydrogen peroxide by bronchoalveolar lavage cells

Freshly isolated BAL cells obtained from rats exposed to the quartz preparations were used to determine spontaneous and PMA-induced *ex vivo *H_2_O_2 _release. H_2_O_2 _generation was measured as described by Pick and Keisari [30]. Therefore, 1.5 × 10^5 ^cells were incubated in 24 well plates in 500 μl HBSS containing 8.5 U/ml horseradish peroxidase (HRP) and 0.28 mM phenol red (PRS solution). Cells were incubated with or without PMA (100 ng/ml) for 4h at 37°C, 5% CO_2_. The reaction was stopped by addition of 10 μl NaOH (1 M), and absorption was read at 610 nm, against a standard curve of H_2_O_2 _in PRS solution.

### Trolox equivalent antioxidant capacity assay

The TEAC (trolox equivalent antioxidant capacity) assay was performed according to Van den Berg et al. [31], with minor modifications. An ABTS (2-2'-azinobis-(-3 ethylbenzothiazoline-6-sulphonate) radical solution was prepared by mixing 2.5 mM ABAP (2,2'-azobis-(-2-amidinopropane)HCl with 20 mM ABTS solution in 150 mM phosphate buffer (pH 7.4) containing 150 mM NaCl. The solution was heated for 10 min at 70°C and, if necessary, diluted to obtain a solution with an absorbance at 734 nm between 0.68 and 0.72. For measuring antioxidant capacity 100 μl of the cell-free BALF was mixed with 900 μl of the ABTS radical solution. Both native and deproteinized (10% TCA) BALF were tested. The decrease in absorbance at 734 nm 5 minutes after addition of the sample was used for calculating the TEAC. Trolox was used as reference compound. The TEAC of the sample is given as the concentration of a trolox solution that gives a similar reduction of the absorbance at 734 nm.

### DNA isolation and analysis of 8-hydroxy-2'-deoxyguanosine by HPLC/ECD

Lung tissue was removed from the animals, chopped into small pieces, aliquots were snap frozen in liquid nitrogen and stored at -80°C until later measurement of 8-OHdG as described previously [23]. Briefly, lung tissue was homogenated and lysed overnight at 37°C in a NEP/SDS solution (75 mM NaCl, 25 mM EDTA, 50 μg/ml proteinase K, 1% SDS). The DNA was dissolved in 5 mM Tris-HCl (pH 7.4) at a final concentration of 0.5 mg/ml. 8-OHdG formation was measured using high performance liquid chromatography with electrochemical detection (HPLC-ECD). Values were expressed as the ratio of 8-OHdG to deoxyguanosine (dG).

### Western blotting

Lung tissue was removed from the animals, chopped into small pieces, aliquots were snap frozen in liquid nitrogen and stored at -80°C. For preparation of whole protein, lung tissue was homogenised within lysis buffer (1% NP-40, 0.5% sodium deoxycholate, 0.1% SDS in PBS) containing freshly added protease inhibitors. Homogenate-lysis buffer-mix was incubated for 30 min on ice and spun at 15.000 g for 20 min at 4°C. Protein concentrations were determined by BioRad-Assay (according to the Bradford method). Samples were electrophorezed at equal protein concentrations (10 μg) in 10% SDS-polyacrylamide gels, and transferred onto nitrocellulose membranes. Non-specific protein binding was blocked with 5% dried milk powder and 0.05% Tween-20 in PBS. Detection of the APE/Ref-1 protein was performed using a monoclonal antibody (1:2000) and an anti-mouse-IgG whole protein HRP-conjugated secondary antibody (1:5000). Blots were reprobed with an antibody against tubulin (1:5000) and a secondary anti-mouse-IgG whole protein HRP-conjugated antibody (1:5000) for protein normalisation. Band formation was visualised using an ECL-reagent/detection system. Quantification was performed by computer-assisted densitometry scanning using a documentation system (Bio-Rad, Germany) with appropriate software (Gel-doc, Bio-Rad, Germany). For each time point, samples of 4 animals per treatment group were quantitated.

### Lung fixation and immunohistochemistry of 8-OHdG and APE/Ref-1

Lungs of five additional animals per treatment group were instilled *in situ *with 4% paraformaldehyde/PBS (pH 7.4) under atmospheric pressure, removed, fixed, dehydrated, and paraffin embedded. Serial sections of lungs were mounted on different slides and stained either for 8-OHdG or APE/Ref-1. For the detection of both antibodies basically the same method was applied, except for additional steps of RNA digestion and DNA-denaturation for the detection of 8-OHdG. Since both antibodies are monoclonal mouse antibodies, horse serum was used to block unspecific binding. The sections were then incubated with a primary antibody against 8-OHdG (1:100) or against APE/Ref-1 (1:500). Detection was performed by incubation with a secondary biotinylated horse-anti mouse antibody (1:200) followed by the streptavidin-biotin-complex according to the manufacturer's protocol. Diaminobenzidine (DAB) was used as a substrate, and the slides were counter stained with hematoxylin. After washing with distilled water, slides were dehydrated and covered in DePex. For the negative control, control sections were incubated with mouse IgG instead of the primary antibodies at the same IgG concentrations. Slides were analysed using a light microscope (Olympus BX60).

### Quantification of 8-OHdG and Ref-1 staining following immunohistochemistry

For quantification of 8-OHdG or APE/Ref-1 five microscopic areas of the left lung lobe of 4 animals per treatment were randomly selected for analysis at an original magnification of × 1000 (oil immersion). Since the staining for 8-OHdG as well as for APE/Ref-1 predominantly occurred within the cell nucleus, in line with the location of the DNA and the action of its repair, all brown (DAB, positive signal) and blue (hematoxylin, negative signal) stained nuclei were counted and expressed as percentage of total cells. In the lungs of the animals that were treated with the non-coated quartz, we observed specific areas with increased an accumulation of inflammatory cells and early indications of tissue remodelling, likely as a result of the non-uniform lung distribution of the quartz-particles after instillation. Therefore, in this treatment group, quantification of each individual section was performed independently for regions with normal lung architecture and focal pathologically altered regions.

### Analysis of expression and subcellular localisation of APE/Ref-1 in representative lung cell lines

In relation to the observed immunohistochemical staining in the lung sections as will be discussed later, APE/Ref-1 protein expression was further evaluated *in vitro*, using Western blotting in the rat cell lines NR8383 and RLE, representing an alveolar macrophage and type II epithelial cell line, respectively [32,20]. NR8383 cells were cultured in F12-K Nutrient Mixture/15% FCS/penicillin/streptomycin, and RLE cells were cultured in Ham's 12 Mixture/5% FCS/penicillin/ streptomycin. Both cell lines were grown until near confluence and nuclear as well as cytosolic proteins were then prepared by the method of Staal et al. [33]. Briefly, cells were harvested by gentle scraping and then lysed by incubation on ice in Buffer A (10 mM Hepes, 10 mM KCl, 2 mM MgCl2, 1 mM DTT, 0.1 mM EDTA containing freshly added protease inhibitors). After 15 min buffer B was added (Buffer A + 10% NP-40), and lysate was centrifuged at 950 g for 10 min. After collection of the supernatant (cytosolic fraction), the pellet containing cell nuclei was resuspended in buffer C (50 mM Hepes, 50 mM KCl, 300 mM NaCl, 0.1 mM EDTA, 1 mM DTT, 10% glycerol containing freshly added protease inhibitors). This nucleic suspension was incubated on ice by agitation for 20 min, followed by centrifugation at 18,000 g for 10 min. Nucleic proteins from this supernatant were collected and stored like the cytosolic proteins at -80°C until analysis. Before analysis of APE/Ref-1 expression by Western Blotting, protein concentration was determined using the BioRad-Assay (according to the Bradford method) and equal protein amounts of 10 μg were loaded.

For an independent evaluation of the subcellular expression of APE/Ref-1 in the RLE cells, also immunocytochemistry was used, as follows: RLE cells were cultured to near confluence on 4-chamber culture slides (Falcon), and immunocytochemistry was performed using the APE/Ref-1 antibody described before followed by a MFP488 goat anti-mouse IgG antibody. Nuclear counter-staining was performed using Hoechst 33342. Slides were analysed using a fluorescence microscope (Olympus BX60) at an original magnification of × 1000.

### Statistical evaluation

Data are expressed as mean ± SD, unless stated otherwise. Statistical analysis was performed using SPSS version 10 for Windows. ANOVA was used to evaluate differences between treatments. Multiple comparisons where evaluated using Tukey's method. A difference was considered to be statistically significant when p < 0.05. Correlation analysis was performed using Pearson's test.

## Results

### Pulmonary inflammation and toxicity

BAL was used to determine inflammation and toxicity in the rat lungs after the different treatments. Treatment of rats with only 22 μg PVNO or 35 μg AL, amounts calculated from the coating efficiency of both substances, showed no effects on any of the studied BAL parameters (data not shown). However, upon instillation of the three different quartz preparations, the total cell number in the BAL was found to be significantly increased only with the non-coated DQ12 (p < 0.001 vs. control, Table 1). The increased cell number as observed with the native quartz was also reflected by an increase in the total number of alveolar macrophages (p < 0.001 vs. control) as well as by the neutrophil number (p < 0.001 vs. control). Analysis of the percentage of neutrophils, revealed a significant increase following treatment with non-coated DQ12 exposure (p < 0.001 vs. control), as well as following treatment with AL-coated DQ12 exposure (p < 0.001 vs. control), but not with PVNO-coated DQ12. However, compared to the non-coated quartz, both coated preparations showed a significantly lower neutrophil percentage (PVNO: p < 0.001, AL: p < 0.01).

**Table 1:** Markers of inflammation and toxicity in bronchoalveolar lavage

	PBS	DQ12	DQ12+PVNO	DQ12+AL
Total cells (× 10^6^)	1.2 ± 0.4	14.0 ± 3.8***	1.6 ± 0.5^###^	3.7 ± 1.2^###^
Total AM (× 10^6^)	0.8 ± 0.1	2.8 ± 0.9***	1.3 ± 0.5^##^	1.7 ± 0.3^#^
Total PMN(× 10^6^)	0.008 ± 0.008	9.1 ± 3.1***	0.1 ± 0.1^###^	1.4 ± 0.5^###^
PMN (%)	0.8 ± 0.5	64.9 ± 8.7***	6.7 ± 6.6^###^	37.1 ± 4.6*** ^##^
TP (μg/ml)	21.35 ± 8.61	76.1 ± 56.05	23.7 ± 7.94	31.23 ± 14.06
LDH (U/l)	12.2 ± 4.5	140.3 ± 38.2***	16.2 ± 5.2^###^	36.8 ± 12.1^###^
AP (U/l)	16.53 ± 2.09	22.15 ± 2.50*	12.82 ± 0.68^###^	15.25 ± 2.04^##^

Significant differences of the particle instilled animals vs. PBS controls are shown by *** p < 0.001; ** p < 0.01; * p < 0.05. Significant differences of the surface-modified quartzes vs. native quartz are shown by ^### ^p < 0.001; ^## ^p < 0.01; ^# ^p < 0.05.

Total protein, LDH and AP were analysed in the BALF to evaluate pulmonary toxicity. None of the treatments showed a significant increase in total protein, although the levels tended to be higher upon treatment with the non-coated quartz. In contrast, quartz-treatment caused a significant increase in LDH (p < 0.001), which could be blocked by both coatings (p < 0.001 compared to DQ12). Similarly, the BALF level of the epithelial toxicity marker AP, which was found to be significantly enhanced by non-coated DQ12 (p < 0.05 compared to control), was found to be reduced by both coatings (PVNO: p < 0.001, AL: p < 0.01).

**Figure 1:**
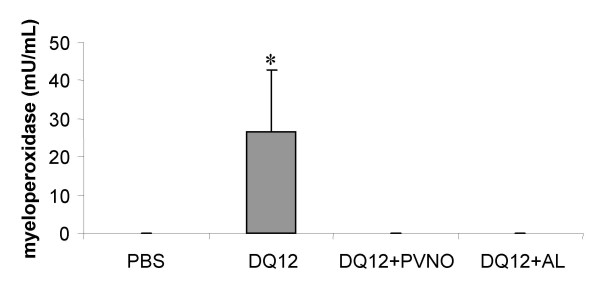
Myeloperoxidase activity in BALF of rat lungs 7 days following i.t. instillation of 2 mg DQ12 or DQ12 coated with either AL or PVNO. Data are expressed as mean ± SD (n = 5). *p < 0.01 vs. PBS (ANOVA, Tukey).

The activity of MPO was determined in BALF to further evaluate the effect of the different particle preparations on neutrophilic inflammation. MPO activity was found to be significantly increased following exposure to the non-coated DQ12 (p < 0.001 compared to control). Coating with PVNO or AL were both able to inhibit this increase (p < 0.001 compared to DQ12, Fig. 1). On a single animal level, covering all treatments, a significant correlation was found between neutrophil numbers and MPO activity (r = 0.639, p < 0.01) confirming the source-specificity of this enzyme.

### Formation of ROS/RNS

**Figure 2:**
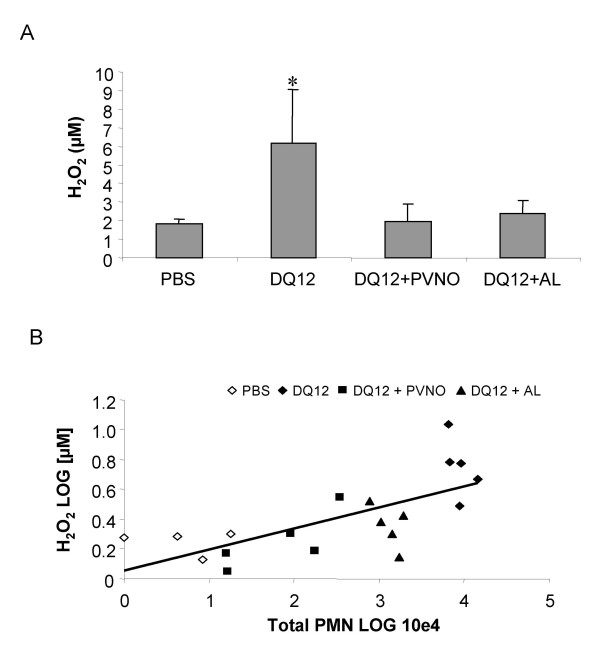
(A) H_2_O_2 _generation in the rat lung. H_2_O_2_levels were analysed spectrophotometrically in the BALF obtained from rats exposed to non-coated DQ12 or DQ12 coated with AL or PVNO (7 days after i.t. instillation). Data are expressed as mean ± SD (n = 5). *p < 0.01 vs. PBS (ANOVA, Tukey). (B) Correlation between H_2_O_2 _levels and total number of neutrophils in the BAL 7 days after i.t. instillation of 2 mg non-coated DQ12 or DQ12 coated with AL or PVNO.

As a relative stable marker for ROS production *in vivo*, H_2_O_2 _levels were determined in BALF obtained from the differently treated animals. Exposure to the native quartz was found to result in a significant increase in the steady-state H_2_O_2 _concentrations (p < 0.05), whereas both coated preparations failed to do so (Fig. 2A). The concentrations of H_2_O_2 _in the BALF were significantly related to the total cell numbers (r = 0.59, p < 0.01). More specifically, BALF H_2_O_2 _was also correlated with the total number of neutrophils (r = 0.62, p < 0.01, see Fig. 2B), but not with the total number of macrophages in the BAL.

**Figure 3:**
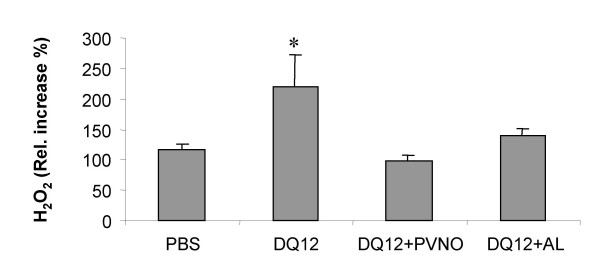
*Ex vivo *release of H_2_O_2 _from PMA-stimulated BAL cells. BAL cells, obtained from rats exposed to non-coated DQ12 or DQ12 coated with PVNO or AL (7 days after i.t. instillation) were cultured *in vitro *(4 h) with or without PMA (100 ng/ml) to activate their oxidative burst. The graph shows the ratio between spontaneous and PMA-induced H_2_O_2 _production, which is expressed as % increase. Data are presented as mean ± SD (n = 3). *p < 0.01 vs. PBS.

In addition, we determined *ex vivo *H_2_O_2 _generation by BAL cells from the different treatment groups upon PMA activation. Data are shown in Fig. 3 and are expressed as relative increase (%) of H_2_O_2_generation in comparison to the cellular H_2_O_2 _generation in the absence of PMA stimulation (spontaneous release). PMA-induced increase in H_2_O_2 _release was found to be significantly elevated with the BAL cells obtained from rats that were treated with native DQ12 (p < 0.05), but not with the cells obtained from animals exposed to the coated quartz preparations.

**Figure 4:**
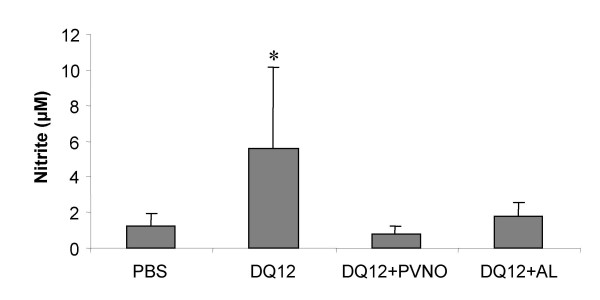
Nitrite levels as detected in the BALF obtained from rats exposed to non-coated DQ12, or DQ12 coated with AL or PVNO (7 days after i.t. instillation). Data are expressed as mean ± SD (n = 5). *p < 0.05 vs. PBS.

In order to determine the generation of nitric oxide in rat lungs following the different particle treatments, levels of its relative stable metabolite nitrite were determined in BALF using the Griess-assay. Animals that were treated with the non-coated DQ12 sample, showed significantly higher BALF nitrite levels indicative of NO production (p < 0.05 compared to the controls), whereas both surface-modified samples did not show any difference from controls (Fig. 4). A significant correlation was found between nitrite levels and the total number of cells in the BAL (r = 0.478, p < 0.05). The correlations between nitrite and total number of macrophages or total number of neutrophils did not reach statistical significance.

### Total non-enzymatic antioxidant capacity

**Figure 5:**
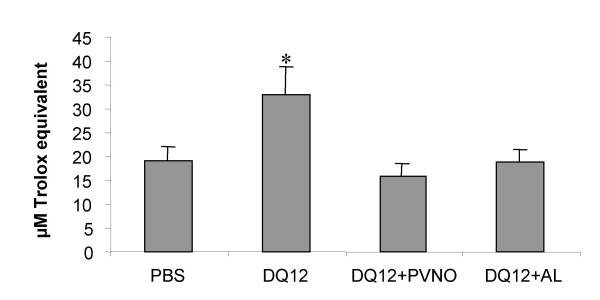
Non-enzymatic total antioxidant capacity (TEAC) of BALF obtained from rats 7 days after i.t. instillation of non-coated DQ12 or DQ12 coated with AL or PVNO. Data are presented as mean ± SD (n = 5). *p < 0.01.

The TEAC assay was used to determine changes in the total non-enzymatic antioxidant capacity of the BALF. Compared with the lavage fluids from the PBS-treated rats, TEAC levels were significantly increased in the BALF from rats treated with native quartz (p < 0.05, Fig. 5), whereas no significant increase could be observed in the lavage fluids from rats exposed to DQ12 from which the surface was coated with either AL or PVNO. No differences in antioxidant capacity was found in the deproteinized BALF (data not shown), suggesting that all the antioxidant capacity was contained within the protein fraction of the BALF.

### Determination of the oxidative stress-induced DNA lesion 8-OHdG in lung tissue

**Figure 6:**
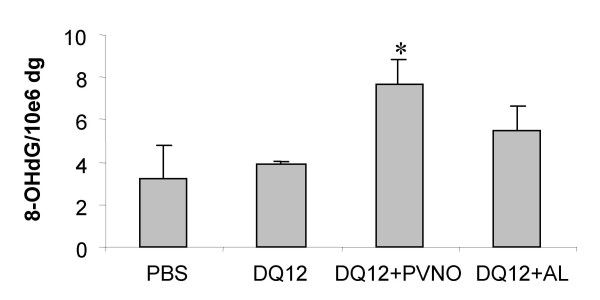
8-OHdG analysis by HPLC/ECD in lung tissue, obtained from rats exposed to 2 mg non-coated DQ12 or DQ12 coated with PVNO or AL (7 days after i.t. instillation). Data are presented as mean ± SD (n = 5). *p < 0.01 vs. PBS.

DNA of whole lung homogenate was investigated for the premutagenic DNA adduct and established oxidative stress marker 8-OHdG by HPLC/ECD [21]. Results of this analysis are shown in Fig. 6. As can be seen in the figure, no enhanced 8-OHdG/dG ratios were observed in the lung homogenates from the animals that were treated with native quartz, whereas surprisingly, these ratios tended to be higher in the lung homogenate DNA from the rats that were treated with the coated quartz preparations. In fact, this increase reached a statistical significance for the PVNO-coated quartz (p < 0.05, Fig. 6).

**Figure 7:**
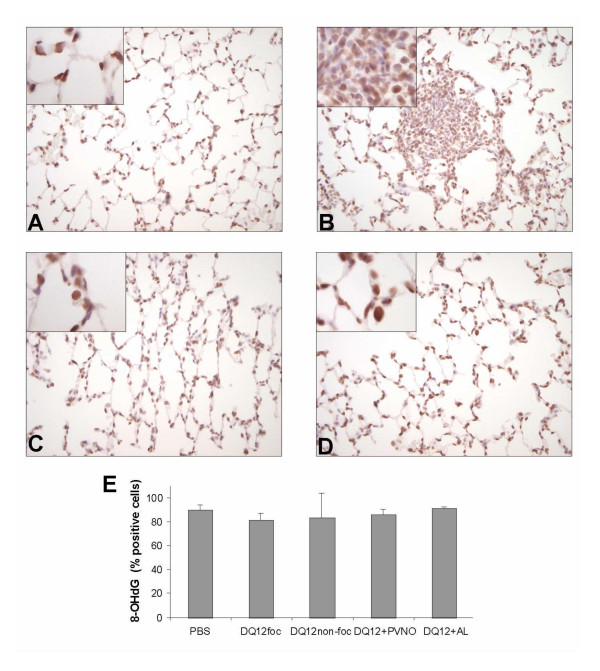
(A-D) Representative images of lung sections, obtained from controls (A) or rats exposed to 2 mg non-coated DQ12 (B) or DQ12 coated with PVNO (C) or AL (D), 7 days after i.t. instillation, stained with an antibody against 8-OHdG (original magnification × 400, original magnification of inserts × 1000). E: Positive cells were quantified in five random chosen areas (n = 4) at a magnification of × 1000.

Using an alternative assay, 8-OHdG was also investigated immunohistochemically in the lung tissue sections. Qualitative visual examination of the staining signal intensity, which appeared to be of a distinct nuclear appearance, did not show any differences between the experimental groups (Fig. 7A–D). Subsequent quantification of the proportion of positive stained nuclei from randomly analysed sections also did not show any difference between the treatments (Fig. 7E). In the animals treated with the non-coated DQ12 multiple focal lesions were observed (Fig. 7B). In order to evaluate whether cellular aggregation might have influenced the results, we performed further analysis in this treatment group, by differentiation between focal and non-focal regions. However, this quantification of 8-OHdG staining did not show any difference between the focal and non-focal regions of this treatment group (Fig. 7E).

### Determination of APE/Ref-1 in lung tissue

**Figure 8:**
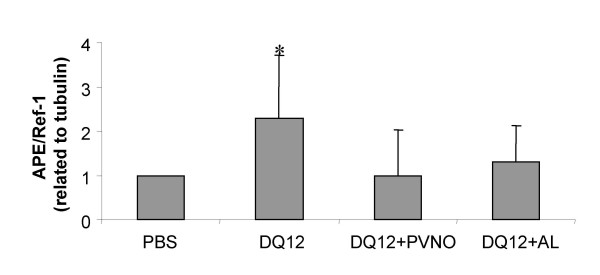
Semi-quantitative analysis of APE/Ref-1 Western blots of lung homogenates of 5 animals per group exposed to PBS, 2 mg non-coated DQ12, or DQ12 coated with PVNO or AL 7 days after a single i.t. instillation. Data are presented as mean ± SD (n = 4). *p < 0.01 vs. PBS.

Whole lung homogenates of the experimental animals were investigated for the expression of the bifunctional APE/Ref-1 protein by Western blotting. Fig. 8 demonstrates the results of densitometry analysis of the APE/Ref-1 expression of 4 animals per treatment. An increase of APE/Ref-1 protein expression was detected in the group instilled with non-coated quartz compared to the control (p < 0.05). Surface modification by PVNO as well as by AL did not show any difference to the control animals.

**Figure 9:**
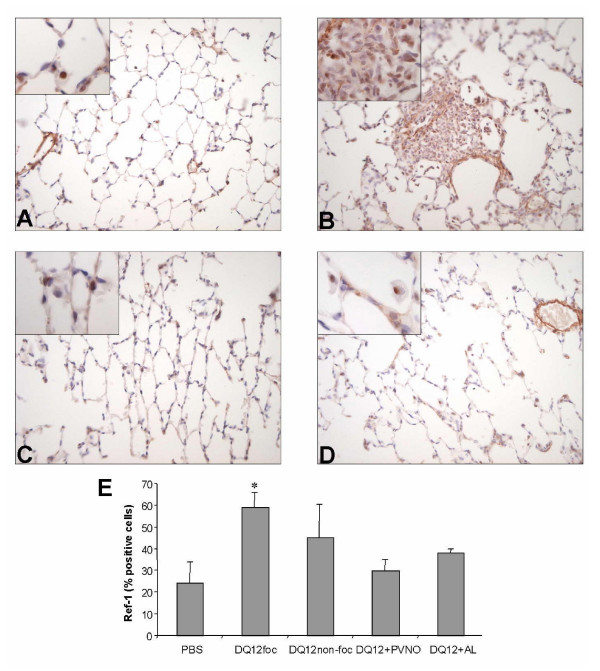
(A-D): Representative images of lung sections, obtained from a control rat (A) or rats exposed to 2 mg non-coated DQ12 (B) or DQ12 coated with PVNO (C) or AL (D), 7 days after i.t. instillation, stained with an antibody against APE/Ref-1 (original magnification × 400, original magnification of inserts × 1000. (E) Positive stained cells were quantified in five random areas of the left lung lobe of four animals per group at a magnification of × 1000.

To confirm these results, and to assess its cellular localisation, lung tissue sections were also analysed for APE/Ref-1 expression using immunohistochemistry. In fact, serial lung sectioning approach was used were tissues, analysed before for 8-OHdG, were investigated with the APE/Ref-1 antibody (Fig. 9A–D). Immunohistochemical imaging analysis revealed a distinct nuclear staining which contrasted with a very weak cytosolic staining in various cell types. This pattern of cytosolic versus nuclear staining seemed to be similar for all treatment groups including the control animals (Fig. 9A–D). Specifically, clear positive nuclear staining signals could be observed within alveolar macrophages as well as within alveolar epithelial cells. The overall APE/Ref-1 expression was shown to be increased in lung sections of animals that were treated with non-coated DQ12 (Fig. 9B versus 9A). Subsequently, we analysed the proportion of positive nuclei using the same approach as followed for 8-OHdG quantification. This counting analysis revealed a significant increase in the % of APE/Ref-1 stained nuclei, specifically in the focal lesions with accumulated cells of the native quartz-group (Fig. 9E). In contrast, no enhanced APE/Ref-1 signal was found in the lung sections of animals that received PVNO- or AL-coated DQ12 (Fig. 9C, D, E).

### APE/Ref-1 expression in rat alveolar epithelial and macrophage cell lines in vitro

**Figure 10:**
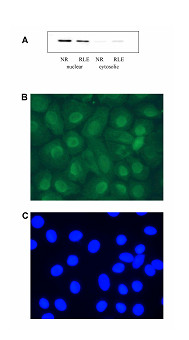
(A) Representative image of Western blot investigation of the expression of APE/Ref-1 protein in nuclear and cytosolic extracts of alveolar macrophages (NR8383) as well as rat lung epithelial cells (RLE) cultured in complete medium. (B-C) Immunocytochemical image of (B) APE/Ref-1 staining and (C) Hoechst nuclear counter-staining in RLE cells. Original magnification of × 1000.

To further validate our *in vivo *observations concerning the apparent alveolar macrophage and epithelial APE/Ref-1 expression, we comparatively evaluated its expression *in vitro *in representative rat cell lines, i.e. NR8383 and RLE. Results of Western blotting analysis of both nuclear and cytosolic protein fractions, revealed a strong nuclear accumulation of APE/Ref-1 in the macrophages as well as in the epithelial cells, whereas in both cell lines only a weak distribution in the cytoplasm was found (Fig. 10A). Reprobing of the blots using an antibody against tubulin, a strong cytoplasmic protein, verified that our nuclear fraction had no cytoplasmic impurities (data not shown). As an independent evaluation of the subcellular distribution pattern of APE/Ref-1 we also performed immunocytochemistry. Results for the RLE cells are shown in Fig. 10B. As can be seen in the figure, this analysis confirmed the predominant nuclear appearance of APE/Ref-1 in these cells. As such, these *in vitro *findings were in concordance with our *in vivo *observations, concerning the predominant nuclear staining pattern.

## Discussion

The data presented in this paper are part of larger ongoing *in vitro *and *in vivo *investigations on the role of surface reactivity in quartz-induced genotoxic, proliferative and fibrogenic effects [10,11,15]. Here we report on the effects of surface modification on quartz-induced generation of ROS and RNS in rat lungs in relation to their involvement in the induction of an oxidative stress response (DNA damage, APE/Ref-1 expression) in the lung tissue. Previously, we and others have shown that coating of quartz-particles with PVNO or AL impairs its ability to elicit pulmonary inflammation (i.e. *in vivo*), as well as the generation of ROS by neutrophils or macrophages *in vitro *[4,9-11,16,17]. In the present study we showed for the first time, that modification of the quartz-surface with PVNO or AL also abrogates *in vivo *formation of ROS and RNS in rat lungs.

Our current observation that exposure to a pure quartz sample (DQ12) causes increased pulmonary levels of ROS and RNS in rat, is in line with earlier studies by others using Min-U-Sil quartz [34,35]. Moreover, we found strong relations between total numbers of phagocytes and RNS-levels as well as between total neutrophil numbers and H_2_O_2_-levels in the rat lungs in relation to the different particle treatments. Together, these observations contribute to the general opinion on the crucial impact of inflammatory cell-related processes in particle-induced lung diseases [36].

For a clear discussion on the role of phagocytes in quartz-related oxidant generation, a distinction between ROS and RNS should be made. With respect to ROS, the present study has focused on the detection of H_2_O_2_, the relative stable dismutation product of superoxide, which is the initial product of the oxidative burst. It has been established that neutrophils are far more potent superoxide-releasing cells than alveolar macrophages [37]. In agreement with this, both PVNO and AL coatings significantly reduced the quartz-induced neutrophil influx as well as H_2_O_2 _levels in the BALF, and both parameters were found to be significantly correlated. In contrast to these observations, previously we showed impaired ROS generation from neutrophils upon *in vitro *treatment with PVNO-coated quartz, but not AL-coated quartz, when compared to treatment with native quartz [10]. This contradiction possibly illustrates that direct particle-cell interactions, as mainly studied using *in vitro *experiments, are of a minor relevance in determining neutrophilic ROS release *in vivo*, i.e. within the lung. It also suggests that surface coatings of quartz primarily affect mechanisms regulating the neutrophil influx into the lung, rather than affecting their subsequent activation. The major contribution of neutrophils as a source of pulmonary H_2_O_2 _is, however, further illustrated by our current *ex vivo *experiments, showing that the only BAL cells from non-coated quartz-treated rats, characterised by the highest proportion of neutrophils, could be significantly activated by PMA.

Apart from ROS, RNS are considered to be a major pool of oxidants that contribute to tissue damage during inflammatory processes. The present study has focused on nitrite, as it is a relative stable metabolite of the initial product NO. In our study the positive correlation between total number of macrophages and nitrite failed to reach statistical significance, suggesting the involvement of an additional cellular source of NO in response to silica, such as alveolar type II epithelial cells (35). In general however, alveolar macrophages are known as the major NO-generating cells in the lower lung, and these cells have been shown to produce much more NO than neutrophils [38]. The major role of alveolar macrophages in particle-related NO-production is even better illustrated by studies from Huffman and colleagues [39], who reported that in response to LPS or silica in rats, up to 100% of NOx produced by BAL phagocytes was derived from alveolar macrophages. Furthermore, it has been demonstrated that exposure to quartz results in a clear increase of iNOS mRNA levels in BAL cells [34,40]. Notably, we (data not shown) and others could not detect any *in vitro *generation of nitrite by quartz-exposed macrophages [41]. Thus it is likely to suggest that the reduction of nitrite levels in the lungs of coated-quartz treated animals mainly results from an inhibited macrophage influx into the lung, rather than from a direct inhibitory effect of coated-quartz on the NO-generation by the macrophages. This also suggests that other factors, including pulmonary cell-cell interactions play a crucial role in activation of NO-release by macrophages per se [41]. This is illustrated by data from Huffman and colleagues [42], who demonstrated that an interaction between macrophages and recruited neutrophils was a crucial factor in the *in vivo *NO-production upon quartz exposure.

Oxidative stress is defined as a disturbance in the balance between production of ROS/RNS and antioxidant defence, in favour of the former, which causes potential damage [43]. Thus in order to assess oxidative stress in our system, apart from determining ROS/RNS production *in vivo*, we also evaluated the *in vivo *antioxidant protection as well as its possibly resulting damage by BAL analysis of toxicity markers and lung tissue induction of 8-OHdG and APE/Ref-1. Silica exposure has been previously demonstrated to cause increased expression and activity of enzymatic antioxidants [44]. In the current study, we applied the TEAC assay to evaluate the effects of quartz on the total non-enzymatic antioxidant capacity of the lung. It was shown that the increase in antioxidant capacity in the lung was most pronounced upon exposure to non-coated quartz, although this was predominantly associated with the protein fraction of the BALF. Nevertheless, in spite of this increased antioxidant protection, clear pulmonary toxicity (i.e. increased LDH and AP levels in BALF) was demonstrated, suggesting an imbalance between generation of ROS/RNS and protective antioxidant pathways. The present data also provide a possible explanation for our earlier observations on the effects of native versus coated quartz on the induction of DNA strand breakage and NFκB-pathway activation in rat lungs [9-11]. In both processes ROS and RNS are considered as crucial mediators of effect [45-47].

To evaluate the impact of the observed *in vivo *ROS/RNS generation in relation to the different quartz-preparations that were applied, 8-OHdG induction in lung tissue DNA was analysed. 8-OHdG represents the best investigated oxidative and premutagenic DNA lesion, and accordingly it has been forwarded and used as a marker of oxidative stress [21]. Previously, we showed that coating of DQ12 with PNVO or AL completely abrogates 8-OHdG induction in human lung epithelial cells *in vitro *[15]. In addition, using an *in vitro *co-incubation model of activated neutrophils and lung epithelial cells, we also demonstrated 8-OHdG induction in epithelial DNA by neutrophil-generated ROS [23]. As such, hypothetically, oxidative DNA damage by quartz in vivo may occur both or either from the surface-reactivity of the quartz particles themselves and/or from phagocyte-derived ROS during inflammation. Importantly however, using two well-established but independent methods (i.e. HPLC/ECD and immunohistochemistry), we did not observe any significant induction of 8-OHdG in our present study by the uncoated, markedly inflammogenic quartz sample. To further complicate this matter, Seiler and colleagues [48] demonstrated, 21 days after a single i.t. instillation of 1.5 mg DQ12, an increase in 8-OHdG immunoreactivity in the DNA of lung alveolar cells, whereas at 3 days after instillation this increase was not detectable. Yamano and colleagues (1995) in contrast showed relatively rapid-increases in 8-OHdG/dG ratios by HPLC/ECD in lung homogenates of rats following i.t. instillation of 10 mg silica. They observed increased 8-OHdG between 1 and 28 days, although significance was only reached between 1 to 5 days after exposure, but not at 7 or 28 days. Several reasons for the discrepancies among these various studies and our current experiments may be given, including the use of different doses and exposure times, as well as the use of differently "hazardous" species or batches of quartz [2]. In relation to the delayed up-regulation of 8-OHdG as observed by Seiler and co-worker [48], it has been suggested that 8-OHdG induction would predominantly occur in proliferating tissue. However, we did observe neither a clear contrast in visual staining intensities of individuals cells nor any difference in the analysed proportion of positively stained cells for 8-OHdG within the focal versus the non-focal regions of individual lung sections of quartz-treated animals, that would support such possibility.

It has also been suggested that HPLC/ECD analysis may lead to artificial induction of 8-OHdG during the extraction and processing of isolated DNA, which might have lead to increased background levels in control animals and thereby obscuring actual occurring differences [15]. However, this would not explain for the significant positive findings by Yamano and colleagues [21] as well as our unexpected effects with the AL-coated, and more specifically the PVNO-coated quartz samples. A possible explanation for these observations might be that, in contrast to the severe inflammation induced by the non-coated quartz, very mild inflammatory responses as observed with the coated samples, fail to up-regulate compensatory feedback mechanisms such as antioxidants and/or oxidative DNA repair actions. At present we are however very cautious about such interpretation, all the more since in our current study the coating-associated effects on 8-OHdG could not be verified by immunohistochemistry.

The hallmark of our observations with regard to the oxidative DNA damage data in current study, was that despite the occurrence of a marked and persistent inflammation, characterised by a 100-fold increased number of neutrophils and a significant increase in pulmonary ROS/RNS levels, no enhanced steady-state expression of 8-OHdG was found by either method of analysis. We therefore hypothesised that quartz-particles and/or their associated inflammatory effects might have caused a compensatory steady-state induction of BER to prevent exponential increases in oxidative DNA damage during conditions of persistent stress. As such, we decided to evaluate the expression of APE/Ref-1, in view of its established *in vivo *inducibility, its redox-sensitivity, as well as its rather broad action-spectrum in ROS mediated damage repair, compared to other BER proteins such as the 8-OHdG glycosylase Ogg-1 [25,49]. As an additional advantage, investigation of repair enzymes, including APE/Ref-1, has the advantage to being oxidation-artefact-free indices of *in vivo*-induced oxidative DNA damage [24].

Our investigations of whole lung homogenate revealed a significant increase in APE/Ref-1 protein expression following instillation of non-coated quartz, but not by the surface-modified quartz samples, in comparison to the control animals. These results were further confirmed by immunohistochemistry, where lung sections from the quartz-treated animals showed increased APE/Ref-1 protein expression in the same lung areas as analysed for 8-OHdG. Subsequent random analysis of the % of positively stained cells showed a significant increase, especially in focal pathologically-altered lung areas. To date, in the field of particle research, the induction of APE/Ref-1 has only been described *in vitro *for asbestos fibres, namely in mesothelial cells and in alveolar macrophages [26,27]. Here we show for the first time that exposure to respirable quartz-dust leads to enhanced expression of APE/Ref-1 in rat lung *in vivo*. With regard to its cell-specificity, intense nuclear staining was observed within alveolar macrophages but also epithelial cells, which are known for their involvement in quartz-induced inflammatory, proliferative, as well as genotoxic effects. In support of these *in vivo *observations, concomitant *in vitro *analysis of APE/Ref-1 expression in NR8383 alveolar macrophages and RLE lung epithelial cells, showed for both cell types a constitutive, predominantly nuclear expression of this protein. Whether the observed *in vivo *effects on APE/Ref-1 expression are driven by possible direct action of the reactive quartz surface or indirectly via action of phagocyte-derived products including ROS or RNS remains to be clarified, and will be a major part of our further *in vitro *investigations. Interestingly in this regard, H_2_O_2 _has recently been described to cause nuclear accumulation and *de novo *synthesis of APE/Ref-1 protein in gastric epithelial cells [40], in a process which could be inhibited by antioxidant pre-treatment. On the other hand, quartz particles may also be directly implicated in line with the *in vitro *observations with asbestos [26,27]. Whereas in the mesothelial cell study, asbestos was reported to enhance both nuclear and mitochondrial APE/Ref-1 expression in relation to its DNA repair actions [26], in the macrophage studies, the observed asbestos effect were connected to its regulation of redox-sensitive transcription [27]. Similarly, apart from current indications for its role in oxidative DNA damage repair, also current *in vivo *observations are indicative for a role of APE/Ref-1 in the well-known proliferative and fibrogenic effects of quartz [19]. First of all, the currently observed immunohistochemical staining patterns for APE/Ref-1 are well in line with our previous work, were we showed that quartz, unlike coated-quartz, causes *in vivo *activation of the NFκB pathway in alveolar macrophages and lung epithelial cells [9,11]. Furthermore, the comparatively stronger nuclear staining of APE/Ref-1 among cells within the focal pathologic lesions compared to non-focal locations as observed 7 days after quartz instillation, also points towards a possible role of this bifunctional protein in quartz-induced proliferation *in vivo*. Accordingly, in our future studies we will further investigate the complex kinetics of APE/Ref-1 expression as a potential hallmark of quartz pathogenesis in relation to its dual involvement, i.e. oxidative DNA damage repair redox-regulation of inflammatory and proliferative signalling pathways.

## Conclusion

The present study showed that coating of the reactive particle surface inhibited quartz-induced production of ROS and RNS in the respiratory tract, a process that was closely associated with a reduced level of inflammatory cells. Obviously, since these endpoints were obtained at the persistent stage of inflammation (i.e. seven days following instillation) one should be cautious to extrapolate our results to the acute inflammatory response by quartz. Pulmonary ROS and RNS release is considered as a crucial and unifying factor in the quartz-induced adverse health effects, including fibrogenicity and carcinogenicity. Despite the fact that non-coated quartz caused a significant ROS/RNS generation and lung tissue damage (i.e. LDH, AP), oxidative DNA damage in the form of 8-OHdG was not increased in the lung. Notably however, the same treatment was found to significantly enhance the expression of APE/Ref-1, a BER pathway protein, also involved in the specific repair of 8-OHdG lesions. On the one hand, our data provide further support that DNA repair enzymes, specifically APE/Ref-1, represent more sensitive and less artefact-prone markers of oxidative stress in models of *in vivo *oxidant exposure than oxidative DNA damage markers, e.g. 8-OHdG. On the other hand, our observations suggest that during quartz-elicited pulmonary inflammation and associated oxidant generation, pathways of oxidative DNA damage repair may be up-regulated to prevent and/or to compensate for the induction and persistence of oxidative DNA damage and possibly resulting mutagenesis. To confirm this hypothesis, our current investigations are focusing on evaluation of the expression of other BER-pathway related proteins apart from APE/Ref-1, as well as on the specific analysis of the actual activity of BER-repair.

## List of abbreviations

2,2'-azinobis-(-3 ethylbenzothiazoline-6-sulphonate) (ABTS), 2,2'-azobis-(-2-amidinopropane)HCl (ABAP), Activator protein 1 (AP-1), alkaline phosphatase (AP), aluminium lactate (AL), apurinic/apyrimidinic endonuclease (APE), base excision repair (BER), bronchoalveolar lavage (BAL), bronchoalveolar lavage fluid (BALF), diaminobenzidine (DAB), dimethyl sulphoxide (DMSO), electron spin resonance (ESR), fetal calf serum (FCS), 8-hydroxy-2'-deoxyguanosine (8-OHdG), Hank's balanced salt solution (HBSS), high performance liquid chromatography with electrochemical detection (HPLC-ECD), horseradish peroxidase (HRPO), intraperitoneal (i.p.), intratracheal (i.t.), lactate dehydrogenase (LDH), May-Grünwald/Giemsa (MGG), myeloperoxidase (MPO), nitric oxide (NO), Nuclear factor kappa B (NFκB), phenol red (PRS solution), phorbol-12-myristate-13-acetate (PMA), phosphate buffered saline (PBS), polyvinylpyridine-N-oxide (PVNO), rat lung epithelial cells (RLE), reactive oxygen and nitrogen species (ROS/RNS), redox effector factor (Ref)-1, trolox equivalent antioxidant capacity (TEAC)

## Declaration of competing interests

The author(s) declare that they have no competing interests.

## Authors' contributions

CA: General study design and supervision (role of surface coating in quartz pathogenesis), instillation and sectioning, immunohistochemical assays and analysis, preparation of manuscript.

AK: Study design (investigations of oxidative stress), developed/conducted *in vivo *and *ex vivo *ROS/RNS assays, preparation of manuscript (equal contributions by CA and AK).

AB: Sectioning, analysis of toxicity markers in BAL

DH: Bronchoalveolar lavage and sectioning, analysis of cellular inflammation in BAL.

PH: Western blotting analyses.

FvS: Contribution to experimental design cf. HPLC/ECD analysis of 8-OHdG.

PB: Initial development and contribution to general study design (role of surface coating in quartz pathogenesis).

RS: Contribution to experimental design, statistics, editing of final version of manuscript.

All authors read and approved the final manuscript.

## References

[B1] IARC (1997). IARC Monograph on the Evaluation of the Carcinogenic Risk of Chemicals to Humans.

[B2] Donaldson K, Borm PJA (1998). The quartz hazard: a variable entity. Ann Occup Hyg.

[B3] Fubini B, Hubbard A (2003). Reactive oxygen species (ROS) and reactive nitrogen species (RNS) generation by silica in inflammation and fibrosis. Free Radic Biol Med.

[B4] Begin R, Masse S, Rola-Pleszczynski M, Martel M, Desmarais Y, Geoffroy M, LeBouffant L, Daniel H, Martin J (1986). Aluminum lactate treatment alters the lung biological activity of quartz. Exp Lung Res.

[B5] Brown GM, Donaldson K, Brown DM (1989). Bronchoalveolar leukocyte response in experimental silicosis: modulation by a soluble aluminum compound. Toxicol Appl Pharmacol.

[B6] Vallyathan V, Kang JH, Van Dyke K, Dalal NS, Castranova V (1991). Response of alveolar macrophages to in vitro exposure to freshly fractured versus aged silica dust: the ability of prosil 28, an organosilane material, to coat silica and reduce its biological activity. J Toxicol Environ Health.

[B7] Vallyathan V, Castranova V, Pack D, Leonard S, Shumaker J, Hubbs AF, Shoemaker DA, Ramsey DM, Pretty JR, McLaurin JL, Khan A, Teass A (1995). Freshly fractured quartz inhalation leads to enhanced lung injury and inflammation. Potential role of free radicals. Am J Respir Crit Care Med.

[B8] Castranova V, Castranova V, Dala NS, Vallyathan V (1995). Suppression of the cytotoxicity and fibrogenicity of silica with PVPNO. Silica and silica-induced lung diseases.

[B9] Duffin R, Gilmour PS, Schins RPF, Clouter A, Guy K, Brown DM, MacNee W, Borm PJA, Donaldson K, Stone V (2001). Aluminium lactate treatment of DQ12 quartz inhibits its ability to cause inflammation, chemokine expression and NF-kappaB activation. Toxicol Appl.

[B10] Knaapen AM, Albrecht C, Becker A, Höhr D, Winzer A, Haenen GR, Borm PJA, Schins RPF (2002). DNA damage in lung epithelial cells isolated from rats exposed to quartz: role of surface reactivity and neutrophilic inflammation. Carcinogenisis.

[B11] Albrecht C, Schins RPF, Höhr D, Becker A, Shi T, Knaapen AM, Borm PJA (2004). Inflammatory time course following quartz instillation: role of TNFα and particle surface. Am J Respir Cell Mol Biol.

[B12] Shi X, Ding M, Chen F, Wang L, Rojanasakul Y, Vallyathan V, Castranova V (2001). Reactive oxygen species and molecular mechanism of silica-induced lung injury. J Environ Pathol Toxicol Oncol.

[B13] Knaapen AM, Borm PJ, Albrecht C, Schins RP (2004). Inhaled particles and lung cancer. Part A: Mechanisms. Int J Cancer.

[B14] Castranova V (2004). Signaling pathways controlling the production of inflammatory mediators in response to crystalline silica exposure: role of reactive oxygen/nitrogen species. Free Radic Biol Med.

[B15] Schins RPF, Duffin R, Höhr D, Knaapen AM, Shi T, Weishaupt C, Stone V, Donaldson K, Borm PJA (2002). Surface modification of quartz inhibits toxicity, particle uptake, and oxidative DNA damage in human lung epithelial cells. Chem Res Toxicol.

[B16] Hedenborg M, Klockars M (1989). Quarz-dust-induced production of reactive oxygen metabolites by human granulocytes. Lung.

[B17] Nyberg P (1991). Polyvinylpyridine-N-oxide and carboxymethyl cellulose inhibit mineral dust-induced production of reactive oxygen species by human macrophages. Environ Res.

[B18] Vallyathan V, Mega JF, Shi X, Dalal NR (1992). Enhanced generation of free radicals from phagocytes induced by mineral dusts. Am J Respir Cell Mol Biol.

[B19] Albrecht C, Borm PJA, Unfried K (2004). Signal transduction pathways relevant for neoplastic effects of fibrous and non-fibrous particles. Mut Res.

[B20] Driscoll KE, Deyo LC, Carter JM, Howard BW, Hassenbein DG, Bertram TA (1997). Effects of particle exposure and particle-elicited inflammatory cells on mutation in rat alveolar epithelial cells. Carcinogenesis.

[B21] Yamano Y, Kagawa J, Hanaoka T, Takahashi T, Kasai H, Tsugane S, Watanabe S (1995). Oxidative DNA damage induced by silica in vivo. Environ Res.

[B22] Nehls P, Seiler F, Rehn B, Greferath R, Bruch J (1997). Formation and persistence of 8-oxoguanine in rat lung cells as an important determinant for tumor formation following particle exposure. Environ Health Perspect.

[B23] Knaapen AM, Seiler F, Schilderman PA, Nehls P, Bruch J, Schins RP, Borm PJ (1999). Neutrophils cause oxidative DNA damage in alveolar epithelial cells. Free Radic Biol Med.

[B24] Rusyn I, Asakura S, Pachkowski B, Bradford BU, Denissenko MF, Peters JM, Holland SM, Reddy JK, Cunningham ML, Swenberg JA (2004). Expression of base excision DNA repair genes is a sensitive biomarker for in vivo detection of chemical-induced chronic oxidative stress: identification of the molecular source of radicals responsible for DNA damage by peroxisome proliferators. Cancer Res.

[B25] Tell G, Damante G, Caldwell D, Kelley MR (2005). The intracellular localization of APE1/Ref-1: more than a passive phenomenon?. Antioxid Redox Signal.

[B26] Fung H, Kow YW, Van Houten B, Taatjes DJ, Hatahet Z, Janssen YM, Vacek P, Faux SP, Mossman BT (1998). Asbestos increases mammalian AP-endonuclease gene expression, protein levels, and enzyme activity in mesothelial cells. Cancer Res.

[B27] Flaherty DM, Monick MM, Carter AB, Peterson MW, Hunninghake GW (2002). Oxidant-mediated increases in redox factor-1 nuclear protein and activator protein-1 DNA binding in asbestos-treated macrophages. J Immunol.

[B28] Klebanoff SJ, Waltersdorph AM, Rosen H (1984). Antimicrobial activity of myeloperoxidase. Meth Enzymol.

[B29] Gallati H, Pracht I (1985). Horseradish peroxidase: kinetic studies and optimization of peroxidase activity determination using substrates H_2_O_2 _and 3,3',5,5'-tetramethylbenzidine. J Clin Chem Clin Biochem.

[B30] Pick E, Keisari YA (1980). Simple colorimetric method for the measurement of hydrogen peroxide produced by cells in culture. J Immunol Meth.

[B31] Van den Berg R, Haenen GRMM, Van den Berg H, Bast A (1999). Applicability of an improved Trolox equivalent antioxidant capacity (TEAC) assay for evaluation of antioxidant capacity measurements of mixtures. Food Chem.

[B32] Helmke RJ, Boyd RL, German VF, Mangos JA (1987). From growth factor dependence to growth factor responsiveness: the genesis of an alveolar macrophage cell line. In Vitro Cell Dev Biol.

[B33] Staal FJ, Roederer M, Herzenberg LA, Herzenberg LA (1990). Intracellular thiols regulate activation of nuclear factor kappa B and transcription of human immunodeficiency virus. Proc Natl Acad Sci U S A.

[B34] Carter JD, Driscoll KE (2001). The role of inflammation, oxidative stress, and proliferation in silica-induced lung disease: a species comparison. J Env Pathol Toxicol Oncol.

[B35] Porter DW, Millecchia L, Robinson VA, Hubbs A, Willard P, Pack D, Ramsey D, McLaurin J, Khan A, Landsittel D, Teass A, Castranova V (2002). Enhanced nitric oxide and reactive oxygen species production and damage after inhalation of silica. Am J Physiol Lung Cell Mol Physiol.

[B36] Greim H, Borm P, Schins R, Donaldson K, Driscoll K, Hartwig A, Kuempel E, Oberdorster G, Speit G (2001). Toxicity of fibers and particles. Inhal Toxicol.

[B37] Kamp DW, Dunn MM, Sbalchiero JS, Knap AM, Weitzman SA (1994). Contrasting effects of alveolar macrophages and neutrophils on asbestos-induced pulmonary epithelial cell injury. Am J Physiol.

[B38] Padgett EL, Pruett SB (1995). Rat, mouse and human neutrophils stimulated by a variety of activating agents produce much less nitrite that rodent macrophages. Immunology.

[B39] Huffman LJ, Prugh DJ, Millecchia L, Schuller KC, Cantrell S, Porter DW (2003). Nitric oxide production by rat bronchoalveolar macrophages or polymorphonuclear leukocytes following intratracheal instillation of lipopolysaccharide or silica. J Biosci.

[B40] Ding SZ, O'Hara AM, Denning TL, Dirden-Kramer B, Mifflin RC, Reyes VE, Ryan KA, Elliott SN, Izumi T, Boldogh I, Mitra S, Ernst PB, Crowe SE (2004). Helicobacter pylori and H2O2 increase AP endonuclease-1/redox factor-1 expression in human gastric epithelial cells. Gastroenterology.

[B41] Castranova V, Huffman LJ, Judy DJ, Bylander JE, Lapp LN, Weber SL, Blackford JA, Dey RD (1998). Enhancement of nitric oxide production by pulmonary cells following silica exposure. Environ Health Perspect.

[B42] Huffman LJ, Judy DJ, Castranova V (1998). Regulation of nitric oxide production by rat alveolar macrophages in response to silica exposure. J Toxicol Environ Health A.

[B43] Sies H (1991). Oxidative stress II. Oxidants and Antioxidants.

[B44] Janssen YM, Marsh JP, Absher MP, Hemenway D, Vacek PM, Leslie KO, Borm PJ, Mossman BT (1992). Expression of antioxidant enzymes in rat lungs after inhalation of asbestos or silica. J Biol Chem.

[B45] Schraufstätter I, Hyslop PA, Jackson JH, Cochrane CG (1988). Oxidant-induced DNA damage of target cells. J Clin Invest.

[B46] Spencer JP, Jenner A, Chimel K, Aruoma OI, Cross CE, Wu R, Halliwell B (1995). DNA strand breakage and base modification induced by hydrogen peroxide treatment of human respiratory tract epithelial cells. FEBS Lett.

[B47] Janssen-Heininger YM, Macara I, Mossman BT (1999). Cooperativity between oxidants and tumor necrosis factor in the activation of nuclear factor (NF)-kappaB: requirement of Ras/mitogen-activated protein kinases in the activation of NF-kappaB by oxidants. Am J Respir Cell Mol Biol.

[B48] Seiler F, Rehn B, Rehn S, Hermann M, Bruch J (2001). Quartz exposure of the rat lung leads to a linear dose response in inflammation but not in oxidative DNA damage and mutagenicity. Am J Respir Cell Mol Biol.

[B49] Fritz G, Grosch S, Tomicic M, Kaina B (2003). APE/Ref-1 and the mammalian response to genotoxic stress. Toxicology.

